# Shame Mediates the Relationship Between Negative Trauma Attributions and Posttraumatic Stress Disorder (PTSD) Symptoms in a Trauma Exposed Sample

**DOI:** 10.32872/cpe.7801

**Published:** 2022-09-30

**Authors:** Rebecca Seah, David Berle

**Affiliations:** 1 Graduate School of Health, University of Technology Sydney, Sydney, Australia; 2 School of Psychiatry, University of New South Wales, Sydney, Australia; Philipps-University of Marburg, Marburg, Germany

**Keywords:** shame, Posttraumatic Stress Disorder, PTSD, negative attributions, trauma

## Abstract

**Background:**

Theoretical models of self-conscious emotions indicate that shame is elicited through internal, stable, and global causal attributions of the precipitating event. The current study aimed to investigate whether these negative attributions are related to trauma-related shame and PTSD symptom severity.

**Method:**

A total of 658 participants aged 18 to 89 (M *=* 33.42; SD *=* 12.17) with a history of trauma exposure completed a range of self-report measures assessing trauma exposure, negative trauma-related attributions, shame, and PTSD symptoms.

**Results:**

Higher levels of internal, stable, and global trauma-related attributions were significantly associated with shame and PTSD. Shame mediated the association between trauma-related attributions and PTSD symptom severity, even after controlling for the effects of number of trauma exposures, worst index trauma and depression.

**Conclusions:**

The present results suggest that negative attributions are a critical cognitive component related to shame and in turn, PTSD symptom severity. Future research should aim to replicate these findings in a clinical sample and extend these findings using prospective designs.

## Highlights

- Cognitive antecedents of shame were investigated in a large trauma-exposed sample.
- Internal, stable, and global trauma attributions were associated with shame severity.
- Trauma-related shame mediated the association between trauma-related attributions and PTSD symptoms.
- Specific attributions may be an important predictor of trauma-related shame.

The exposure to a potentially traumatic event (PTE) often elicits a myriad of emotional responses that intensify traumatic stress reactions. Moreover, these reactions are thought to contribute to the development and maintenance of current threat characteristic of posttraumatic stress disorder (PTSD). Recently, there has been a growing interest in the role of shame as an important emotional trauma sequalae linked to poorer adjustment and maladaptive coping and predictive of the development of PTSD symptoms (e.g., intrusive recollections, hyperarousal and avoidance) (Saraiya & Lopez-Castro, 2016).

The cognitive model of PTSD offers a framework for understanding how shame may emerge following exposure to PTEs (Ehlers & Clark, 2000; Elwood et al., 2009). According to the model, the nature of the emotional responses in persistent PTSD varies according to appraisals of the trauma and its sequalae (Ehlers & Clark, 2000). For example, the model posits that appraisals concerning attributions of responsibility and perceived violation of internal and societal standards may evoke feelings of shame.

In line with this, theoretical models of shame classify it as a self-conscious emotion, as it arises when the self is implicated by a negative and aversive event that violates internal and/or external standards and evokes judgement from others (Gilbert, 1997; Lewis, 1971; Tangney & Dearing, 2002). Specifically, shame is said to arise through a cognitive-evaluative process, where the eliciting event is attributed to internal, stable, and global attributions; causes that relate to aspects of the individual that are present across all situations and likely to affect situations across one’s life (e.g., one’s character) (Lewis, 1971; Tangney & Dearing, 2002; Tracy & Robins, 2004). Guilt, which also arises from internal attributions, is distinct from shame in that the attribution pertains to a specific action (unstable) which does not affect all situations (Specific) (e.g., one’s behaviour). This subtle difference in cognitive attributions is important as guilt and shame prompt distinct responses. The phenomenological experience of shame is the desire to withdraw and hide due to perceived judgement from others and threat of being exposed (Gilbert, 2000). In contrast, guilt tends to prompt behavioural responses that are motivated by reparative efforts.

Indeed, higher levels of internal, stable and global attributions has been associated with higher levels of PTSD. While, these studies have focused on negative attributional style, which is the tendency to attribute events to internal, stable and global causes to common negative and/or hypothetical life events (Elwood et al., 2009), PTEs can be considered phenomenologically distinct to general negative life events and exert greater influence on current PTSD symptoms (Gray & Lombardo, 2004; Reiland et al., 2014).

Following exposure to a traumatic event, posttraumatic shame may arise through this appraisal process, where the individual erroneously blames themselves for having caused the event. Consequently, the self is implicated in an unwanted event, and the trauma and its effects are appraised as having occurred due to the individual being inadequate or worthless in some way. Even in the absence of an external threat, the individual may still feel a sense of impending threat due to fear of rejection and stigmatisation but also an internal threat due to ongoing negative self-evaluation. Consequently, feelings of trauma related shame are likely to be painful, prompting avoidance that inhibits trauma processing, which impedes recovery (Leonard et al., 2020). For example, in their conceptual model of shame and adjustment in child sexual abuse survivors, Feiring et al. (1996) proposed that shame arises from sexual abuse through the mediation of cognitive attributions and that such shame in turn leads to poorer overall adjustment. A number of studies of child sexual abuse survivors have reported findings consistent with this model as well as the possibility that shame may mediate the relationship between negative attributions and PTSD symptom severity (Alix et al., 2017; Feiring et al., 2002; Uji et al., 2007). Although promising, these studies utilised abuse specific attributions and shame measures which limit their generalisability to other trauma exposed populations. Further, the attribution measure did not explicitly assess the dimensions of internal, stable and global attributions which is considered a necessary component of the attribution-emotion link to shame (Lewis, 1971; Tangney & Dearing, 2002; Tracy & Robins, 2004).

Although negative attributions are purported to be a cognitive antecedent to shame, there are several trauma characteristics that may impact the severity of posttraumatic cognitions and emotions. Firstly, although trauma exposure is insufficient to elicit trauma related shame, the nature of the traumatic event may function as a diathesis toward making more negative appraisals and higher levels of shame. For example, individuals with interpersonal trauma exposure, defined as an event that involves deliberate perpetration of harm to another individual (e.g., sexual assault, armed robbery, physical threats etc.) (Forbes et al., 2014) have reported increased levels of shame and PTSD (La Bash & Papa, 2014). In a recent study, Zerach and Levi-Belz (2018) found that experiencing a morally injurious event may contribute to an increased tendency to make internal, stable and global attributions, trauma related shame and more severe posttraumatic stress symptoms (PTSS). Their findings indicate that it is possible that certain trauma types may increase one’s tendency to make negative attributions, subsequently eliciting higher levels of shame.

Secondly, routine self-report PTSD screening measures require a single designated trauma event to be used in assessing the severity of symptoms. However, the exposure to multiple potentially traumatic events can be considered a rule not the exception. There is robust evidence indicating that, with an increased number of PTE exposures, PTSD risk increases in a dose-dependent manner (Tortella-Feliu et al., 2019). Also, the severity of PTSD symptoms increases when participants are asked to rate symptoms across their trauma history (Simpson et al., 2011). Furthermore, the potential effect of time elapsed since the indexed trauma event may also impact endorsement of self-conscious cognitions and emotions (Bryant et al., 2017). Thus, consideration of the cumulative impacts of PTEs along with time since trauma exposure is pertinent.

Regardless of overall trauma exposure, it is expected that individuals will seek to assign meaning and provide causal attributions to explain their experiences. Thus, the current study sought to extend previous findings in two ways. Firstly, it aimed to investigate the relationships between trauma specific negative attributions (higher internal, stable, and global attributions) shame and PTSD symptom severity in a broad sample of trauma exposed survivors. Based on previous findings, it was hypothesised there would be significant associations between negative attributions, shame, and PTSD symptoms. Secondly, it explored whether trauma-related shame would mediate the relationship between higher levels of internal, stable, and global attributions on the one hand, and PTSD symptoms on the other.

To examine the unique contributions of trauma related attributions and shame in relation to PTSD, the current study controlled for the effects of the various trauma characteristics mentioned. This included cumulative lifetime exposure to PTEs, reference trauma type (interpersonal vs. non-interpersonal) and time elapsed since reference trauma. Symptoms of depression were also controlled for due to depression’s significant comorbidity with PTSD (Flory & Yehuda, 2015). It was hypothesised that even after controlling for these covariates, trauma-related shame would mediate the relationship between attributions and PTSD symptom severity.

## Method

### Participants

Six hundred and sixty-seven participants consented to participate in the study, however nine participants failed the attention checks, and were excluded from the analyses. The final sample consisted of 658 participants between the ages of 18 to 89 (*M =* 33.42; *SD* = 12.17) who consented to participate in the study. A majority (*n* = 257; 39.1%) of the sample resided in the United States, with a similar proportion from the United Kingdom (*n* = 249; 37.8%). The sample consisted of 346 women (52.6%), 300 men (45.6%) and 12 (1.9%) preferring to self-describe. Just over half the participants (*n* = 371; 56.4%) reported being in a relationship or were married, 258 (39.2%) had never been married and 29 (4.4%) were either separated or divorced. Slightly under half (*n* = 206; 31.3%) of participants disclosed at least one mental health disorder diagnosis from a professional. Among those who chose to specify, 223 (*n* = 33.9%) reported a current diagnosis of depression and/or anxiety. 80 participants reported currently seeking mental health support from a healthcare professional. Just over half of participants’ (58.7%) self-reported PTSD symptoms placed them within the clinical range for a provisional PTSD diagnosis (PCL-5 total scores ≥ 31) (Bovin et al., 2016).

Participants endorsed exposure to an average of 6.3 (*SD =* 2.2) potentially traumatic events (PTE) across their lifetime. In terms of type of trauma exposure, transportation accidents (*n =* 406; 61.7%), severe life-threatening illnesses (*n =* 227; 34.5%), and unwanted/uncomfortable sexual experiences, including sexual assault (*n* = 209; 31.8%) were the most common trauma categories endorsed. The most common reference trauma endorsed was some form of direct exposure (personally experienced and/or witnessed it happening to a close family member/friend) to an interpersonal trauma (e.g., physical and/or sexual assault and psychological abuse) (*n* = 219; 33.3%), followed by some form of transport accident (*n* = 154; 23%), and various forms of illnesses and/or physical injury (*n* = 109; 16.7%). The mean elapsed time since the reference trauma was 11.6 years (*SD* = 10.7).

### Measures

#### The Lifetime Events Checklist (LEC)

The LEC (Weathers, Blake, et al., 2013b) is a 17-item self-report measure used to screen for exposure to potentially traumatic events (PTE) in a respondent’s lifetime. It consists of 16 known events and an additional item assessing any stressful life events not listed. Respondents indicate their level of exposure for each PTE on a 6-point nominal scale. Following this, participants are asked to identify and briefly describe the worst event they experienced, specifically the event that they classify as the most distressing. This event was used as the reference trauma for assessing current symptoms of PTSD. The LEC does not yield a total composite score. The LEC demonstrated adequate psychometric properties as a stand-alone measure for trauma exposure (Gray et al., 2004). In the current study, a total lifetime trauma load was calculated by summing the number of traumatic experiences across each type of trauma endorsed by the individual.

#### The PTSD Checklist for DSM-5 (PCL-5)

The PCL-5 (Weathers, Litz, et al., 2013) a 20-item self-report questionnaire which was administered to assess PTSD symptoms. Participants endorse the extent to which they were bothered by PTSD symptoms in relation to their reference trauma in the past month (e.g., “Repeated disturbing and unwanted memories of the stressful experience”) on a 5-point Likert Scale, 0 (*Not at all*) to 4 (*Extremely*). A total symptom severity score was obtained by summing each item, with a score higher than 31 indicating the presence of probable PTSD (Bovin et al., 2016). The PCL-5 has demonstrated strong reliability and validity and is psychometrically sound instrument for quantifying PTSD symptom severity (Bovin et al., 2016).

#### The Expanded Attributional Style Questionnaire - Trauma (EASQ-T)

The EASQ (Peterson & Villanova, 1988) is a measure used to assess a respondent’s tendency to generate specific attributions for hypothetical aversive events. Participants are asked to rate the cause of each event. On this scale, respondents are asked to rate the cause of each event on 7-point Likert scale for three dimensions; 1) Internal or External (“Is the cause something about you or about other people and/or circumstances”), 2) Stable or Unstable (“In the future, will this cause be present?”) and 3) Specific or Global (“Is this cause something that affects just this type of situation or does it influence other aspects of your life?”). The EASQ has previously demonstrated adequate to good internal consistencies (Peterson & Villanova, 1988).

The EASQ was adapted by Reiland et al. (2014) to assess trauma related attributions. On the EASQ-Trauma, participants rate the cause of each traumatic event they were exposed to according to the LEC (Weathers, Blake, et al., 2013b) on the EASQ dimensions of Internal-External, Stable-Unstable and Specific-Global. The score on each attribution dimension ranged between 1 and 7. An overall attribution score or negative trauma score was calculated by averaging the sum of each dimension. Higher overall scores on the scale indicate higher levels of internal, stable and global attributions.

#### The Trauma Related Shame Inventory (TRSI)

The TRSI (Øktedalen et al., 2014) is a 24-item measure of trauma related shame. Respondents rate the extent that they experience thoughts and feelings associated with shame in relation to their traumatic experiences over the past week on a 4-point Likert Scale, 0 (*Not true of me*) to 4 (*Completely true of me*). Sample items include “Because of what happened, I am disgusted with myself”, “If others knew what happened to me, they would be ashamed”. A total trauma-related shame score was computed by summing all items on the TRSI. The TRSI has demonstrated strong content and construct validity and discriminate validity from the Trauma Related Guilt Inventory (Kubany et al., 1996).

#### The Depression Anxiety and Stress Short Form Scale (DASS-21)

The DASS-21 (Lovibond & Lovibond, 1995) is a widely used screening measure of distress in both clinical and non-clinical settings. It consists of 21 items comprised of three self-report scales of depression, anxiety, and stress symptoms. In the current study, only the 7-item depression subscale was used to yield a total depression score. Respondents endorse the extent to which they experienced symptoms over the past week on a 4-point Likert scale, 0 (*Did not apply to me at all*) to 4 (*Applied to me very much, or most of the time*). A total depression score was computed by summing all the items on the depression subscale. The DASS-21 has demonstrated good discriminant validity relative to other depression measures and high internal consistency (Henry & Crawford, 2005).

### Procedure

Participants were recruited from Australia, Canada, Ireland, The United Kingdom and United States via Prolific Academic (ProA), an online crowdsourcing platform. Only participants over the age of 18 and who endorsed being exposed to at least one potentially traumatic event (PTE) within their lifetime according to the LEC (Weathers, Blake, et al., 2013b) were included in the study. Participants were administered a battery of self-report questionnaires which assessed their lifetime exposure to PTEs, along with their attributions for these events, trauma related shame, PTSD symptoms and symptoms of depression and anxiety.

### Statistical Analyses

Spearman’s rank order correlations were calculated given the non-normal positively skewed distributions of depression, PTSD, and trauma-related shame. Bootstrapping (5,000) iterations were performed to test the indirect effects of shame and negative attributions in relation to PTSD symptom severity using conditional process analysis (Hayes, 2017). Trauma exposure, depression symptoms, worst reference trauma type, and time since worst reference trauma were entered as covariates.

The use of bootstrapping, a non-parametric resampling method offers an advantage over the traditional Sobel Test as it does not require the assumption of normality to be met for the product of co-efficients. Further, the resampling methods minimises bias that arises from non-normal sampling distributions (Hayes, 2017). Indirect effects are significant when the 95% Confidence Interval (CI) does not contain zero.

## Results

### Univariate and Bivariate Statistics

Mean, standard deviation and range of all self-reported measures are reported in Table 1. The internal consistency for all scales was excellent. All measures were significantly and positively correlated with each other and small to moderate in magnitude (Table 2).

**Table 1:** Means, Standard Deviations, and Reliability of Measures

Variable	*M*	*SD*	Range	Cronbach’s α
Exposure (LEC)	6.31	2.16	2-16	–
Depression (DASS-21)	6.67	6.26	0-21	.94
Internal attributions (EASQ-T Internal)	2.45	1.43	1-7	–
Stable attributions (EASQ-T Stable)	3.73	1.63	1-7	–
Global attributions (EASQ-T Global)	3.34	1.56	1-7	–
Attributions (EASQ-T Total)	3.17	1.07	1-6.58	–
Shame (TRSI)	14.33	15.94	0-70	.97
PTSD (PCL-5)	27.78	19.59	0-80	.95

*Note*. Exposure = Total lifetime trauma exposure to distinct trauma types; Depression = Depression symptoms; Internal, stable and global = internal, stable and global trauma-related attributions; Attributions = Total trauma related attributions; Shame = Trauma Related Shame; PTSD = PTSD symptoms.

**Table 2:** Spearman’s Rank Order Correlations Between Trauma Exposure, Depression Symptoms Trauma-Related Attributions, Trauma Related Shame, PTSD Symptoms

Variable	1	2	3	4	5	6	7	8
1. Exposure	–	.16**	.10**	.10**	.08	.13**	.30**	.19**
2. Depression		–	.27**	.16**	.14**	.31**	.59**	.56**
3. Attributions			–	.54**	.73**	.79**	.27**	.29**
4. Internal				–	.04	.23**	.25**	.20**
5. Stable					–	.42**	.04	.08*
6. Global						–	.35**	.37**
7. Shame							–	.66**
8. PTSD								–

*Note. N* = 587. Exposure = Total lifetime trauma exposure to distinct trauma types (LEC); Depression = Depression symptoms (DASS-21); Attributions = Total trauma related attributions (EASQ-T Total); Internal, stable, and global = internal, stable and global trauma-related attributions (EASQ-T subscales); Shame = Trauma Related Shame (TRSI); PTSD = PTSD symptoms (PCL-5).

*p < .05. **p < .01.

### Mediation Analysis

**Figure 1:**
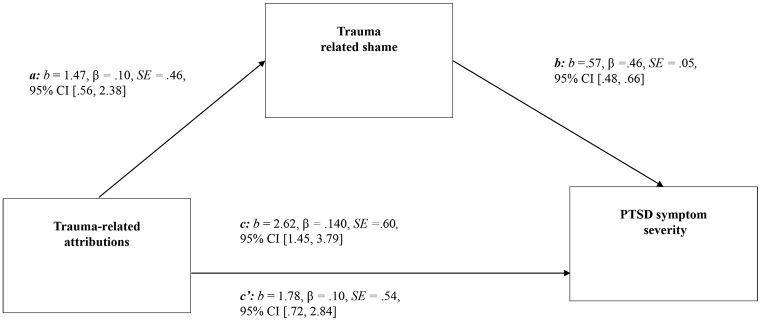
The Relationship Between Trauma-Related Attributions and PTSD Symptom Severity Mediated by Trauma-Related Shame *Note.* c = total effect; c’ = direct effect; *b* = non-standardised regression coefficient; β = standardised regression coefficient; *SE* = standard error; CI = confidence interval. Indirect effect = 95% CI = [.35, 1.34].

Figure 1 reports the results of the bootstrapped mediation analysis. Together, after controlling for lifetime trauma exposure, depression symptoms, worst trauma type, and time since worst trauma, negative attributions and trauma-related shame accounted for significant variance in PTSD symptom severity, *F*(6,652) = 107.53, *R*^2^ = .50, *p* < .001. Trauma related negative attributions exhibited significant direct effects on shame, *b* = 1.47, *p* < .001, 95% CI [.56, 2.38], and shame also had a significant direct effect on PTSD symptoms, *b* = .57, *p* < .001, 95% CI [.48, .66]. Trauma related attributions exhibited a significant indirect effect on PTSD symptoms via shame, 95% CI [.35, 1.34].

However, when trauma related shame was included in the model, the direct effect of trauma-related attributions remained significant, *b* = 1.78, *p* < .001, 95% CI [.72, 2.84], indicating that trauma-related shame partially explains the relationship between trauma-related casual attributions. Thus, it is likely that there are additional mediators that could contribute to the understanding the effect of negative trauma attributions and PTSD symptoms.

As a secondary exploratory analysis, we repeated the mediation analyses for each separate attribution dimension. The results of these are presented in Figures 1-3 in the Supplementary Materials. In brief, both internal, 95% CI [.53, 1.30] and global, 95% CI [.32, 1.10] attributions exhibited significant indirect effects on PTSD symptoms via shame. In contrast, there was no significant indirect effect for stable attributions, 95% CI [-.65, .03].

## Discussion

To our knowledge, this is the first study that examines the role of internal, stable and global trauma-related attributions in relation to shame and PTSD symptoms in a broad trauma exposed sample. The purpose of the study was two-fold. Firstly, it aimed to investigate the relationship between negative attributions (higher levels of internal, stable, and global attributions), trauma-related shame and PTSD. Secondly, it investigated whether trauma-related shame would mediate the relationship between negative trauma-related attributions and PTSD symptoms.

As predicted, negative attributions, that is, higher levels of internal, stable, and global attributions and trauma-related shame both had significant direct effects on PTSD symptom severity. Interestingly, although cumulative trauma exposure is an important risk factor for PTSD (Tortella-Feliu et al., 2019), correlation analysis of the present data indicated that the relationship between trauma load and PTSD is negligible. These findings are consistent with both empirical and theoretical evidence implicating maladaptive cognitive appraisals and subsequent emotional reactions as important predictors of PTSD beyond trauma exposure (Cromer & Smyth, 2010; Ehlers & Clark, 2000).

The finding that internal, stable and global attributions are significantly associated with higher levels of PTSD is consistent with previous research indicating strong associations between negative causal attributions and PTSD symptoms (Gómez de La Cuesta et al., 2019). The attribution that one’s experiences are due to internal causes that are unchanging, and pervasive in all domains of life is likely to increase expectancy that future events would reoccur and engender feelings of helplessness and loss of control over life events and one’s future (Mikulincer & Solomon, 1988). Indeed, a sense of helplessness has been associated with a perception of ongoing threat and perceived lack of safety among domestic violence survivors (Salcioglu et al., 2017). Moreover, findings from neuroimaging studies have indicated that cognitive distortions are linked to PTSD through intense re-experiencing of the trauma memory elicited by trauma related cues (Berman et al., 2018; Daniels et al., 2011).

As our findings indicate, negative attributions of the traumatic event were associated with higher levels of trauma-related shame which in turn, were associated with more severe PTSD symptoms. Thus, the appraisal that negative events are due to internal, stable and global attributions may lead to the focus of evaluation being directed inward where the self and its entirety is judged negatively, prompting feelings of intense shame. The cross-sectional nature of our study precludes causal inferences; however, further prospective studies of these variables should seek to confirm this possibility.

The phenomenological experience of shame is painful, motivating the desire to withdraw and hide due to the fear of rejection or stigmatisation. In this way, feelings of shame may increase the intensity of PTSD symptoms through responses such as avoidance (Feiring et al., 2002; Leonard et al., 2020), a core symptom of PTSD that maintains overgeneralised fear and inhibits new learning (Craske et al., 2008). Indeed, a recent study indicates that experiential avoidance may be one of the key mechanisms that explains the relationship between shame and PTSD symptoms (Leonard et al., 2020). However, future research will be needed to bolster such findings. In addition, current theoretical models of shame indicate that feelings of shame are also avoided due to their association with the event and trauma related cues (Lee et al., 2001; Wilson et al., 2006). Consequently, the inability to process shame is likely to intensify these feelings where, in the absence of physical danger, feelings of shame become a source of internal threat.

Although the current results support our second hypothesis, there may be other variables that influence and explain the relationship between shame and PTSD. Following trauma exposure, shame is typically accompanied by other emotional responses such as fear, guilt, alienation, and betrayal that also promote avoidance and intense reliving of trauma memories (Dewey et al., 2014; Held et al., 2015). Moreover, there may be other attributional processes such as perceived controllability and importance of events (Tracy & Robins, 2006) that may be relevant to shame worth investigating.

Overall, the findings support the assertion that individual variability in trauma attributions and reactions are linked to not only an increase in PTSD symptom severity, but this relationship can also be explained by emotional and behavioural reactions associated with shame related to one’s traumatic experiences.

Some limitations of the current study should be noted as avenues for future research. First, the use of a cross-sectional design precludes any causal inferences. It is likely that both negative appraisals and trauma related shame have a bi-directional relationship, however the extent to which they reinforce each other remains an empirical question. Thus, longitudinal research is needed to assess the directionality of these constructs. Second, although the use of self-report questionnaires is common in clinical psychology research, responses may be influenced by participants’ introspective ability and other response biases. Third, additional demographic data was not obtained with respect to ethnicity, or employment status which may be important risk factors for PTSD (Tortella-Feliu et al., 2019). Also, not all participants in our sample were in the clinical range for PTSD, limiting the generalizability of our results to clinical populations.

Fourth, the construct validity of the “global” dimension of the EASQ may be imperfect in that the global dimension items appeared to assess attributions about the perceived consequences of traumas, rather than attributions about the cause itself (“Is this cause something that affects just this type of situation, or does it also influence other areas of your life”). This may have contributed to the relatively stronger associations observed between global attributions and PTSD symptoms when compared with the internal-external and stable-variable dimensions. Future studies should ideally use interviewer-based approaches to allow careful distinctions between attributions about the causes versus the consequences of trauma events.

Further, although the PCL-5 is widely accepted and utilised within trauma research as a PTSD symptom screening tool, it does not examine trauma relatedness of symptoms and significant overlap between PTSD and other psychiatric symptoms may inadvertently inflate PTSD symptom severity scores (Monson et al., 2008). It is worth noting that an individual can make multiple attributions for a single event, especially when the event consists of multiple, closely related events. In the attempt to account for multiple lifetime exposures, we assessed attributions for all PTE exposures. However, an individual can have multiple exposures to the same type of traumatic event, complicating the identification of the particular event that a given attribution corresponds to. Thus, assessment of the index trauma event to assess event specific attributions using a clinician administered diagnostic assessment tool is warranted. For example, the Clinician-Administered PTSD Scale for DSM-5 (CAPS5; Weathers, Blake, et al., 2013a) could be used to identify the index trauma and assess specific attributions in accordance with the event. Further, the use of a diagnostic interview can provide a more accurate diagnostic picture of PTSD symptoms and increase the generalizability of current findings to clinical samples.

Although specific attribution dimensions may exert greater influence on shame and PTSD symptoms than others, the results indicate that, together, internal, stable and global attributions for lifetime exposure to PTEs functions as a potential cognitive vulnerability toward trauma related shame. Thus, targeting these cognitions may constitute an important mechanism for trauma recovery. Cognitive based interventions that utilise attribution retraining such as Cognitive Processing Therapy (CPT; Resick & Schnicke, 1992) has been found to be useful in modifying self-blaming attributions and PTSD (Resick et al., 2002). Moreover, there is some indication that gradual exposure to and processing of trauma memories can significantly reduce shame based cognitive distortions (Cohen et al., 2004). More recently, there has been increasing interest and empirical support for the use of compassion-based therapies are a potential adjunct to existing cognitive interventions for PTSD in facilitating the effectiveness of cognitive reappraisal strategies (Au et al., 2017).

Overall, the present study indicates that following exposure to a PTE, negative attributions are associated with shame, which in turn is associated with higher levels of PTSD symptoms. The findings underscore the potential clinical utility of assessing negative attributions as a potential antecedent of shame. In doing so, clinicians can seek to target these processes and potentially change the trajectory of shame responses and reduce the emotional impact of the trauma and the severity of PTSD symptoms.

## Supplementary Materials

The Supplementary Materials contain the following items (for access see Index of Supplementary Materials below):

- Figure 1 – The relationship between Internal Attributions and PTSD Symptom Severity mediated by Trauma-Related Shame.
- Figure 2 – The relationship between Stable Attributions and PTSD Symptom Severity mediated by Trauma-Related Shame.
- Figure 3 – The relationship between Global Attributions and PTSD Symptom Severity mediated by Trauma-Related Shame.

10.23668/psycharchives.8184 Supplement 1 Supplementary materials to "Shame mediates the relationship between negative trauma attributions and posttraumatic stress disorder (PTSD) symptoms in a trauma exposed sample"

SeahR.
BerleD.
 (2022). Supplementary materials to "Shame mediates the relationship between negative trauma attributions and posttraumatic stress disorder (PTSD) symptoms in a trauma exposed sample"
[Additional figures]. PsychOpen. 10.23668/psycharchives.8184
PMC966733936398006

## Data Availability

Participants in the present study did not consent for their data to be shared publicly, so supporting data for the present study is not available.
