# Supplementary material for: Shame Mediates the Relationship Between Negative Trauma Attributions and Posttraumatic Stress Disorder (PTSD) Symptoms in a Trauma Exposed Sample
Source: Clin Psychol Eur. 2022 Sep 30;4(3):e7801. doi: 10.32872/cpe.7801 (PMC9667339; doi:10.32872/cpe.7801)
Supplement: Supplement 1 [file cpe-04-7801-s01.pdf]

Shame mediates the relationship between negative trauma attributions and posttraumatic stress disorder (PTSD) symptoms in a trauma exposed sample.

Rebecca Seah[1] and David Berle[1,2]

1. Graduate School of Health, University of Technology Sydney, Sydney, Australia

2. School of Psychiatry, University of New South Wales, Sydney, Australia

Author Note

Rebecca Seah https://orcid.org/0000-0003-4724-1568

David Berle https://orcid.org/0000-0002-4861-2220

Manuscript published in Clinical Psychology in Europe: https://doi.org/10.32872/cpe.7801

Corresponding author information:

A/Prof David Berle
Discipline of Clinical Psychology
Graduate School of Health
University of Technology Sydney
PO Box 123, Broadway
NSW 2007, Australia
E: David.Berle@uts.edu.au
P: +61 2 9514 4278

Supplementary Material

Figure 1

The relationship between Internal Attributions and PTSD Symptom Severity mediated by Trauma-Related Shame.

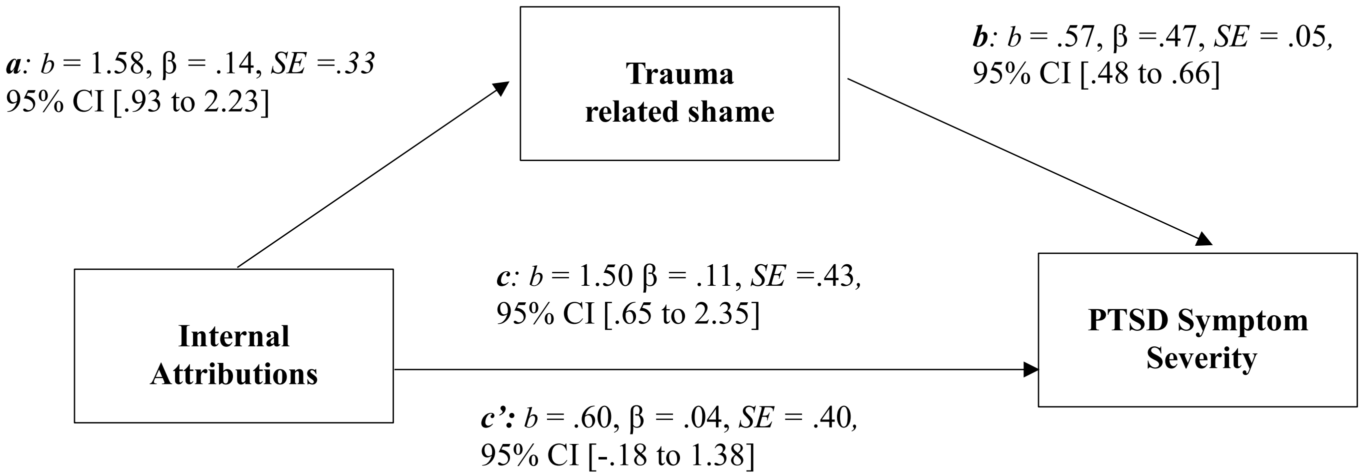

Note. c = total effect; c' = direct effect; *b* = non-standardised regression coefficient; β = standardised regression coefficient; *SE* = standard error; *CI* = confidence interval.

Indirect effect = 95%CI [.53 to 1.30].

Figure 2

The relationship between Stable Attributions and PTSD Symptom Severity mediated by Trauma-Related Shame.

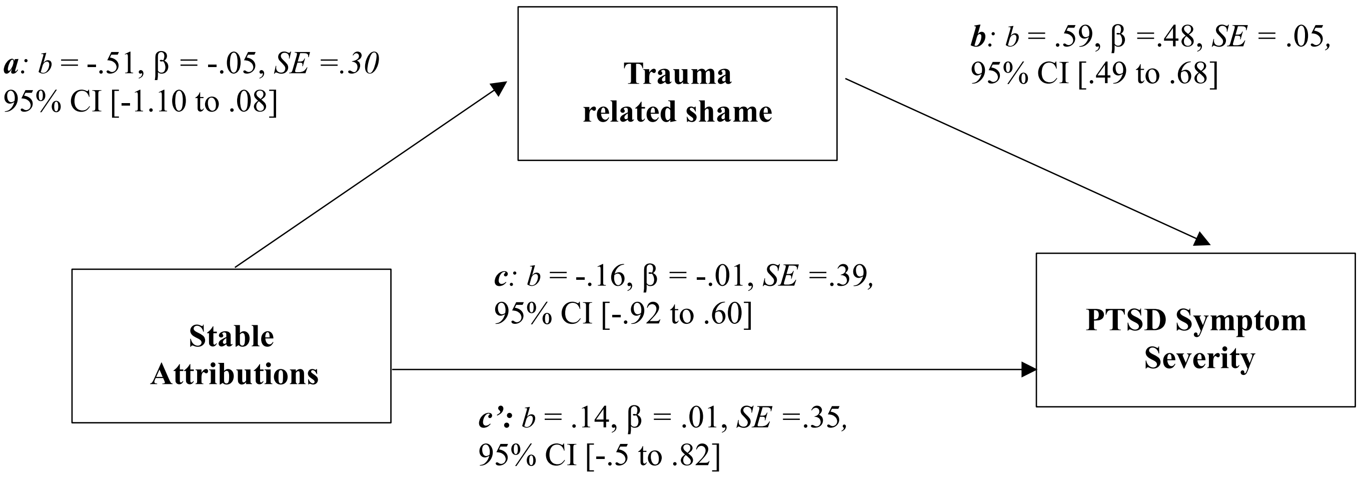

Note. c = total effect; c' = direct effect; *b* = non-standardised regression coefficient; β = standardised regression coefficient; *SE* = standard error; *CI* = confidence interval.

Indirect effect = 95%CI [-.65 to .03].

Figure 3

The relationship between Global Attributions and PTSD Symptom Severity mediated by Trauma-Related Shame.

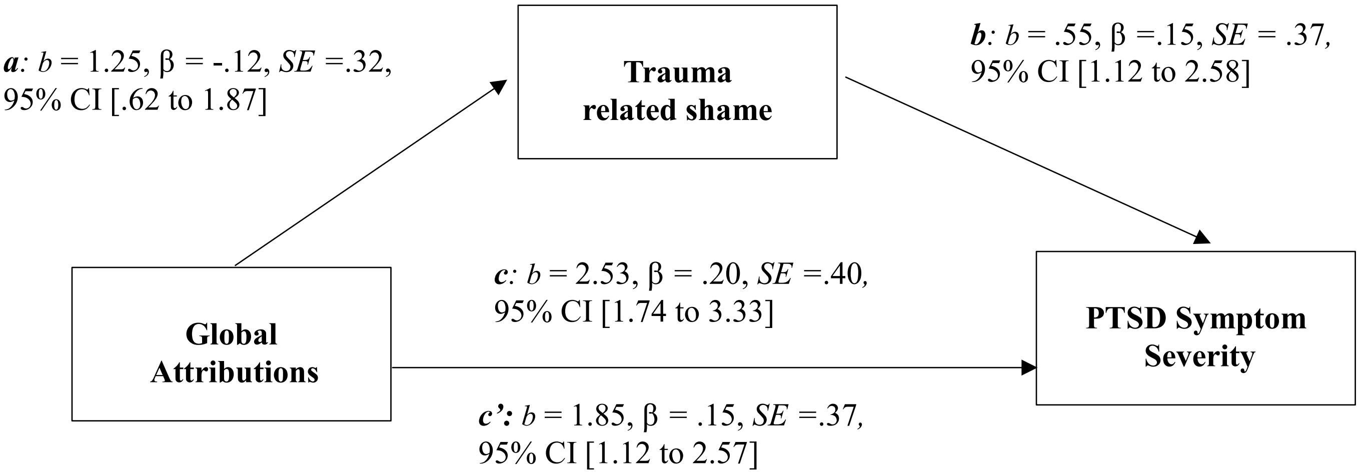

Note. c = total effect; c' = direct effect; *b* = non-standardised regression coefficient; β = standardised regression coefficient; *SE* = standard error; *CI* = confidence interval.

Indirect effect = 95%CI [.32 to 1.10].
